# Deletion of the *ATP2* Gene in *Candida albicans* Blocks Its Escape From Macrophage Clearance

**DOI:** 10.3389/fcimb.2021.643121

**Published:** 2021-04-16

**Authors:** Yishan Zhang, Chuanyan Tang, Zhanpeng Zhang, Shuixiu Li, Yajing Zhao, Luobei Weng, Hong Zhang

**Affiliations:** ^1^ Department of Dermatology, The First Affiliated Hospital of Jinan University, Guangzhou, China; ^2^ Institute of Mycology, Jinan University, Guangzhou, China

**Keywords:** *Candida albicans*, *ATP2* gene, macrophage, host-pathogen interaction, alternative carbon source, glyoxylate cycle

## Abstract

Macrophages provide the first-line defense against invasive fungal infections and, therefore, escape from macrophage becomes the basis for the establishment of *Candida albicans* invasive infection. Here, we found that deletion of *ATP2* (*atp2*Δ/Δ) in *C. albicans* resulted in a dramatic decrease from 69.2% (WT) to 1.2% in the escape rate *in vitro*. The effect of *ATP2* on macrophage clearance stands out among the genes currently known to affect clearance. In the normal mice, the *atp2*Δ/Δ cells were undetectable in major organs 72 h after systemic infection, while WT cells persisted *in vivo*. However, in the macrophage-depleted mice, *atp2*Δ/Δ could persist for 72 h at an amount comparable to that at 24 h. Regarding the mechanism, WT cells sustained growth and switched to hyphal form, which was more conducive to escape from macrophages, in media that mimic the glucose-deficient environment in macrophages. In contrast, *atp2*Δ/Δ cells can remained viable but were unable to complete morphogenesis in these media, resulting in them being trapped within macrophages in the yeast form. Meanwhile, *atp2*Δ/Δ cells were killed by oxidative stress in alternative carbon sources by 2- to 3-fold more than WT cells. Taken together, *ATP2* deletion prevents *C. albicans* from escaping macrophage clearance, and therefore *ATP2* has a functional basis as a drug target that interferes with macrophage clearance.

## Introduction

*Candida albicans* is the most prevalent lethal fungal pathogen and can cause invasive infections in immunodeficiency patients, with variable rates from 5% to 70% (Bassetti et al., 2018; Pappas et al., 2018). The current antifungals target a limited number of cellular processes, and therefore, the development of different therapeutic approaches is urgently needed (Bassetti et al., 2018).

Macrophages provide the first-line defense against invasive fungal infections, and thus, *C. albicans* escape from macrophage becoming the basis for establishing systemic infection (Qian et al., 1994; Lionakis et al., 2013; Ngo et al., 2014; Weiss and Schaible, 2015). During the clearance, macrophages restrict the growth of and destroy *C. albican*s mainly by nutrient deprivation, a low pH, and oxidative stress in the phagosome (Austermeier et al., 2020; Williams and Lorenz, 2020). Furthermore, macrophages secrete cytokines to recruit more phagocytes to participate in pathogen clearance (Ngo et al., 2014). In contrast, similar to other successful intercellular pathogens (*Mycobacterium tuberculosis, Histoplasma capsulatum*, etc.), *C. albicans* has evolved elegant strategies to evade macrophage killing (Weiss and Schaible, 2015; Shen and Rappleye, 2020). These strategies can be divided into the following two categories: strategies that support pathogen survival within macrophages, including rapid conversion to metabolize alternative carbon sources, adaptation and neutralization of acidic phagosomes, and resistance to oxidative stress; and strategies that facilitate macrophage destruction, such as morphogenesis (Uwamahoro et al., 2014; Westman et al., 2019; Shen and Rappleye, 2020; Williams and Lorenz, 2020).

Transcriptomics and proteomics studies have identified some genes that affect the escape of *C. albicans* from macrophages (Lorenz et al., 2004; Kitahara et al., 2015; O’Meara et al., 2018). However, knocking out these genes associated with specific pathways may increase the clearance rate, but it does not completely block the escape of *C. albicans* from macrophages (Danhof et al., 2016; Jain et al., 2018; Williams and Lorenz, 2020). For example, knocking out genes related to non-glucose carbon source utilization or hyphae formation in *C. albicans* can only increase the clearance by only 20-40%, but blocking several pathways simultaneously has additive effects (Piekarska et al., 2006; Danhof and Lorenz, 2015; Jain et al., 2018; Williams and Lorenz, 2020). Therefore, searching for drug targets involved in the macrophage clearance process requires finding genes with a broader range of functions.

Oxidative phosphorylation is the central molecular node of the cellular metabolic network (Marcet-Houben et al., 2009). Here, we found that deletion of *ATP2*, which encodes the β subunit of F_1_F_o_-ATP synthase, greatly reduced the ability of *C. albicans* to escape macrophages *in vitro* and *in vivo*. Regarding the mechanism, the *ATP2* deletion impaired the adaptation of *C. albicans* to glucose-deficient environment within macrophages, and although they could remain viable for a short period of time, they were unable to form hyphae to escape from macrophages and were more susceptible to oxidative stress killing. Due to the high involvement of *ATP2* in host-pathogen interactions, confirming that whether *ATP2* can be used as a drug target deserves further investigation.

## Materials and Methods

### Strains, Cells and Culture Conditions

*C. albicans* strains used in this study were SC5314 (WT, ATCC) and *ATP2* null mutant (*atp2*Δ/Δ) (Li et al., 2018). Strains were maintained on YPD (1% yeast extract, 2% peptone, 2% dextrose) agar at 30°C and stored in 50% glycerol at -80°C. For each assays, *C. albicans* cells were cultured in YPD liquid medium overnight at 30°C and 150 rpm, then collected, washed and normalized to appropriate concentrations.

Murine macrophage-like cell line RAW264.7 (ATCC) was used for all macrophage assays. Cells were maintained in Dulbecco’s modified Eagle medium (DMEM) supplemented with 10% heat-inactivated fetal bovine serum, 100 U/ml penicillin and 100 μg/ml streptomycin, at 37°C with 5% CO_2_.

### Macrophage Killing Assay

The end-point dilution assay was performed to detect the killing effect of macrophages on *C. albicans* as described previously (She et al., 2013). RAW264.7 cells were seeded into 96-well plates at a density of 1×10^5^ cells/well and adhered for 1h. Then 2×10^5^ cells/well *C. albicans* were added and cocultured for 6, 12, 24 and 48 h. At each time point, the co-cultures were treated with 0.1% Triton X-100 for 2 min. A serial dilution was performed and plated onto YPD agar. Numbers of colony-forming units (CFU) were counted after 48 h incubation and the macrophage killing rate was determined by comparing cocultures to *C. albicans* control cultures without macrophages. Assays were performed in triplicate, and experiments were repeated three times.

### Macrophage Cytotoxicity Assay

*C. albica*ns-induced macrophage damage was assessed by measuring the release of lactate dehydrogenase (LDH) using a Non-Radioactive Cytotoxicity assay (Abcam) (She et al., 2013). RAW264.7 (1×10^5^ cells) were co-incubated with *C. albicans* (1×10^5^ cells) for 2, 4, 6, and 8 h. At each time point, the absorbance at 490 nm was recorded according to the manufacturer’s protocol. The release of LDH relative to maximum LDH release was then calculated and corrected for spontaneous release of LDH by the *C. albicans* or macrophages alone. The experiment was performed in triplicate.

### Macrophage Phagocytosis Assay

Activated RAW264.7 cells were seeded in 96-well plates at 1×10^6^ cells/ml (She et al., 2013). *C. albicans* cells were stained with 1.25 mM fluorescein isothiocyanate (FITC, Sigma) for 15 min, then diluted to 2 × 10^6^ cells/ml in RPMI medium and cocultured with macrophages for 30, 90, 120 min. At each time point, 100 μl supernatant was removed and 100 μl trypan blue was added to quench the fluorescence of unengulfed strains. The fluorescence signal intensity (E_x_/E_m_=495/525) of each well were detected with a microplate reader (Thermo Fisher Scientific). Assays were performed in triplicate, and experiments were repeated three times.

### Fungal Burden in Clodronate Liposome Treated Mice

10-week-old female BALB/c mice (20 g) were injected intraperitoneally with 200 μl of clodronate- or PBS-liposome (http://clodronateliposomes.org) both 24 h before and 24 h after intravenous *C. albicans* infections (Wirnsberger et al., 2016). Mice were infected with 2×10^5^ CFU of *C. albicans* cells intravenously. At 24 and 72 h after infection, organs were taken out and weighed, then grinded and measured fungal burden (CFU/g) by the end point dilution assay. There were three mice in each group, and experiment was repeated twice.

### microPET/CT Scanning and Radioimmuno-γ Counting

10-week-old female BALB/c mice (20 g) were infected with 1× 10^6^ CFU of *C. albicans* cells intravenously. Mice were fasted for 12 h before the imaging time point and only water was provided. Each mouse was injected 1% pentobarbital (15 ul per g mouse) intraperitoneally and 5 μCi/g of [^18^F]FDG intravenously 45 min before imaging. PET and CT images were obtained by the Inveon micro-PET/CT (Siemens) small-animal PET imagers with a static acquisition time of 15 min (Davis et al., 2009). After PET/CT scan, the *ex vivo* biodistribution of organs was detected by γ-counter (Perkin-Elmer) (Rolle et al., 2016). There were three mice in each group, and experiments were repeated three times.

### RNA Extraction and Real‐time Quantitative PCR

Total RNA from co-cultured macrophages were extract using Trizol (Invitrogen), and total RNA from *C. albicans* were extract using the E.Z.N.A. Yeast RNA kit (Omega Bio-Tek) (Li et al., 2017). Approximately 0.8 μg of RNA was used to synthesize cDNA (Qiagen, Venlo, Netherlands). RT-qPCR was done in triplicate as previously described using Bio-Rad iQ5. Primers used are shown in **Table S1**. The expression levels of *C. albicans* genes were normalized to 18S rRNA levels, while the macrophage genes were normalized to GAPDH levels. The 2^–ΔΔCT^ (where CT is the threshold cycle) method was used to determine the fold change in gene transcription. Each sample was performed in triplicate and experiments were repeated three times.

### Growth Curve and Cell Viability Assay

*C. albicans* cells were collected and washed with PBS, then inoculated in 100 ml of macrophage-mimicking media (YNB liquid medium supplemented with 2% glucose, CAA, GLcNAc, oleic acid, or lactate) with an initial OD_600_ of 0.02. Shake cultures were grown at 30 °C and OD_600_ of each strain was measured every 2 h (Li et al., 2018). Cell viability was detected by the end-point dilution assay as describe previously. *C. albicans* cells were washed and resuspended in media mentioned above to a density of 1×10^6^ cells/ml. Cultures were grown at 30 °C and 100 μl of liquid was taken out to perform end-point dilution assay every 2 h. All experiments were performed in triplicate and repeated three times.

### Hyphal Morphogenesis of Phagocytosed *C. albicans* Cells

RAW264.7 cells (1×10^6^ cells) were seeded to glass coverslips and incubated overnight (Vylkova and Lorenz, 2014). 500 nM MitoTracker Deep Red FM (Molecular Probes) was added and incubated for 30 min. *C. albicans* cells were stained with FITC as mentioned before, and cocultured with macrophages for 3 h. Images were taken using a confocal laser scanning microscope (Carl Zeiss LSM880), under the set of E_x644_/E_m655_ (macrophages) and E_x488_/E_m525_ (*C. albicans*). The percentage of hyphae cells were counted manually (the number of germinated cells versus the number of total cells). At least 50 cells per strain were counted. Experiments were repeated three times.

### Hyphae Formation Assay

The hyphae formation assay was performed as described previously (Li et al., 2017). *C. albicans* cells were washed and resuspended in YNB liquid medium supplemented with 2% glucose, CAA, GLcNAc, oleic acid, or lactate to a density of 1×10^5^ cells/ml. Cells were incubated in 24 well plates at 37°C for 3 h and images were taken by an inverted microscope (Olympus IX81).

### H_2_O_2_ Killing Assay

The end-point dilution assay was performed to detect the killing effect of H_2_O_2_ on *C. albicans* (Wu et al., 2018). *C. albicans* cells were washed and resuspended in YNB liquid medium supplemented with 2% glucose, CAA, GLcNAc, oleic acid, and lactate to a concentration of 5×10^4^ cells/ml. Then, 6 mM H_2_O_2_ was added except for the growth control well and incubated at 37°C for 6 h. A serial dilution was performed and plated onto YPD agar. CFU were counted after 48 h incubation and the mortality rate was determined by comparison with fungi recovered without exposure to H_2_O_2_.

### Protein Extraction and LC-MS/MS Analysis

*C. albicans* protein was extracted by a liquid nitrogen grinding method as described previously (Kitahara et al., 2015). *C. albicans* cells were diluted into 1 L cultures at a starting OD = 0.5, cultured for 8 h at 30°C, and harvested at 4 °C. The samples were ground into cell powder, then lysis buffer (8 M urea, 1% Triton-100, 10 mM dithiothreitol, and 1% protease inhibitor) was added, followed by sonication three times on ice using a high-intensity ultrasonic processor (Scientz).

The proteins were reduced with 5 mM dithiothreitol for 30 min at 56°C and alkylated with 11 mM iodoacetamide for 15 min. The sample was diluted with urea, and trypsin was added at the mass ratio of trypsin: protein = 1:50 for the first overnight digestion and 1:100 for a second 4 h digestion. The tryptic peptides were fractionated by high pH reverse-phase HPLC using a Betasil™ C18 column (5 μm particles, 250 mm length, Thermo Scientific). The protein digests were then labeled with a Tandem Mass Tag kit (Thermo Scientific) (Kitahara et al., 2015). The peptides were desalted using a Strata X C18 SPE column (Phenomenex) and vacuum-dried. Then the peptides were separated into 60 fractions using a gradient of 8%–32% acetonitrile (pH 9.0) over 60 min. Finally, the peptides were combined into 10 fractions and dried by vacuum centrifugation.

The detailed protocol for LC‐MS/MS was described previously (Herrero-de-Dios et al., 2018; Truong et al., 2019). The tryptic peptides were dissolved in 0.1% formic acid (solvent A) and loaded on an analytical column (75 µm × 150 mm). The constant flow rate was set at 500 nL/min with the step gradients of mobile B (0.1% formic acid in 90% acetonitrile): 9%–26% for 38 min, 26%–35% for 14 min, 35%–80% for 4 min, and held at 80% for 4 min. The peptides were subjected to NSI source followed by tandem MS analysis in Q ExactiveTM Plus (Thermo) coupled online to the UPLC. The mass range was 400–1500 m/z, and tandem mass spectra were recorded in high sensitivity mode. In each cycle, a maximum of 20 precursors were selected for fragmentation, with the dynamic exclusion for 15 s. Protein identification and quantification were performed *via* the Maxquant search engine (v.1.5.2.8). The data were searched against a protein sequence database downloaded from UniProtKB for *C. albicans* SC5314 (total 6040 entries). The quantitative method was set to TMT-10plex, and the FDR for protein identification and PSM identification was adjusted to < 1% (Truong et al., 2019). The mass spectrometry proteomics data have been deposited to the ProteomeXchange Consortium *via* the PRIDE (Perez-Riverol et al., 2019) partner repository with the dataset identifier PXD024039.

### Ethics Statement

All BABL/c mice were obtained from Guangdong Medical Laboratory Animal Center, Foshan, Guangdong, China. The animal experiments were performed in accordance with the recommendations in the Guide for the Care and Use of Laboratory Animals of the National Academy of Science. The protocol was carried out with permission from the Laboratory Animal Ethics Committee of Jinan University (NO. 2019670).

## Results

### Deletion of *ATP2* in *C. albicans* Decreases Its Escape From Macrophages *In Vitro*

**Figure 1:**
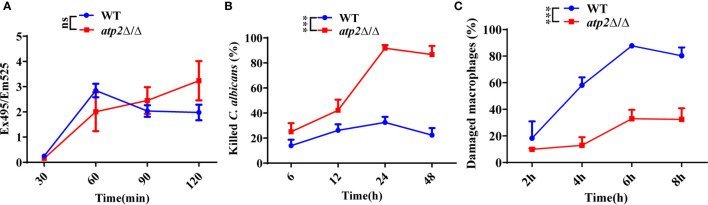
*ATP2* modulates the *C. albicans*-macrophage interaction *in vitro*. **(A)** The phagocytosis of FITC-labeled WT and *atp2*Δ/Δ cells by macrophages was evaluated by measuring FITC fluorescence (Ex495/Em525) in macrophages. **(B)** Percentage of *C. albicans* cells killed by macrophages over time. WT and *atp2*Δ/Δ cells were cocultured with RAW264.7 cells at a ratio of 2:1 (MOI of 2), and the killing rate was determined by comparison with *C albicans* recovered without RAW264.7 cells at each time point. **(C)** Percentage of *C. albicans*-induced macrophage damage was assessed by measuring the release of LDH at each time point. In **(A–C)**, assays were performed in triplicate. Data are shown as mean ± s.d. ***P < 0.001; ns, not significant; by two-way ANOVA.

Escape from macrophage clearance is the basis by which *C. albicans* establishes systemic infection (Lionakis et al., 2013; Ngo et al., 2014; Weiss and Schaible, 2015). To investigate the impact of *ATP2* on this pivotal procedure, we examined the proportion of *C. albicans* (WT and *atp2*Δ/Δ) killed by macrophages (RAW264.7) in a coculture system *via* the end-point dilution assay. When *C. albicans* and macrophages were cocultured at a ratio of 2:1 (multiplicity of infection [MOI] of 2), the *atp2*Δ/Δ and WT cells were phagocytosed at comparable rates (*P* > 0.05) (**Figure 1A**), but more *atp2*Δ/Δ cells were killed by macrophages and less macrophages were damaged at each time point than WT cells (*P* < 0.05) (**Figures 1B, C**). The mortality rate of *atp2*Δ/Δ was 98.2% at 24 h, but only 32.5% in the WT at this time point (*P* < 0.05) (**Figure 1B**). Then we compared the CFUs of WT and *atp2*Δ/Δ in medium without macrophages and found that *atp2*Δ/Δ grew only 31.1% less than WT at 24 h (**Figure S1**). This suggests that the deletion of *ATP2* in *C. albicans* resulted in a significant reduction in the ability to escape macrophage clearance.

### Deletion of *ATP2* in *C. albicans* Decreases Its Escape From Macrophages *In Vivo*

**Figure 2:**
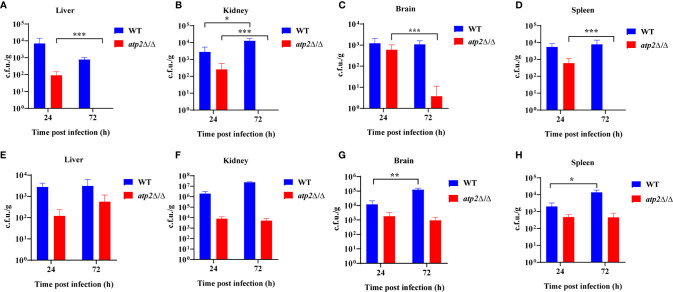
*ATP2* is required for *C. albicans* to resist macrophage clearance *in vivo*. **(A–D)** Fungal burden in the liver **(A)**, kidney **(B)**, spleen **(C)**, and brain **(D)** of mice injected with PBS liposomes 24 h before and 24 h after intravenous infection with *C. albicans* (2×10^5^ CFU per mouse). **(E–H)** Fungal burden in the liver **(E)**, kidney **(F)**, spleen **(G)**, and brain **(H)** of mice injected with clodronate liposomes 24 h before and 24 h after intravenous infection with *C. albicans* (2×10^5^ CFU per mouse). Each group includes 3 mice. In **(A–H)**, data are shown as mean ± s.d. *P < 0.05, **P < 0.01, ***P < 0.001; by Student’s *t*-test.

To further study the effect of *ATP2* on the escape of *C. albicans* from macrophage clearance *in vivo*, we constructed macrophage normal/depleted mice with PBS/clodronate liposome, and detected the fungal burden at different time points after systemic infection with WT or *atp2*Δ/Δ. The results showed that the WT cells were not cleared in the normal mice (PBS liposome), and that the fungal burden in the kidney at 72 h was higher than that at 24 h post-infection (*P* < 0.05); however, the fungal burden in the liver, spleen and brain did not significantly differ from that at 24 h (*P* > 0.05). In contrast, the *atp2*Δ/Δ mutant cells were almost completely cleared 72 h after infection, with no detectable pathogens in the liver, kidney and spleen. Cells remained in the brain were far less than WT (**Figures 2A–D**) (*P* < 0.05).

However, in the macrophage-depleted mice (clodronate liposome), the *atp2*Δ/Δ cells stably persisted in the liver, kidney, spleen, and brain at 24 h and 72 h after infection instead of being cleared. The number of WT cells was increased in the spleen (**Figure 2H**) and brain (**Figure 2G**) but unchanged in the liver (**Figure 2E**) and kidney (**Figure 2F**) 72 h post-infection compared with those at 24 h. These results suggest that *C. albicans* cells lacking *ATP2* were unable to resist macrophage clearance *in vivo*.

### Clearance of *ATP2* Mutant Cells Does Not Result in Increased Recruitment of Phagocytes *In Situ*

**Figure 3:**
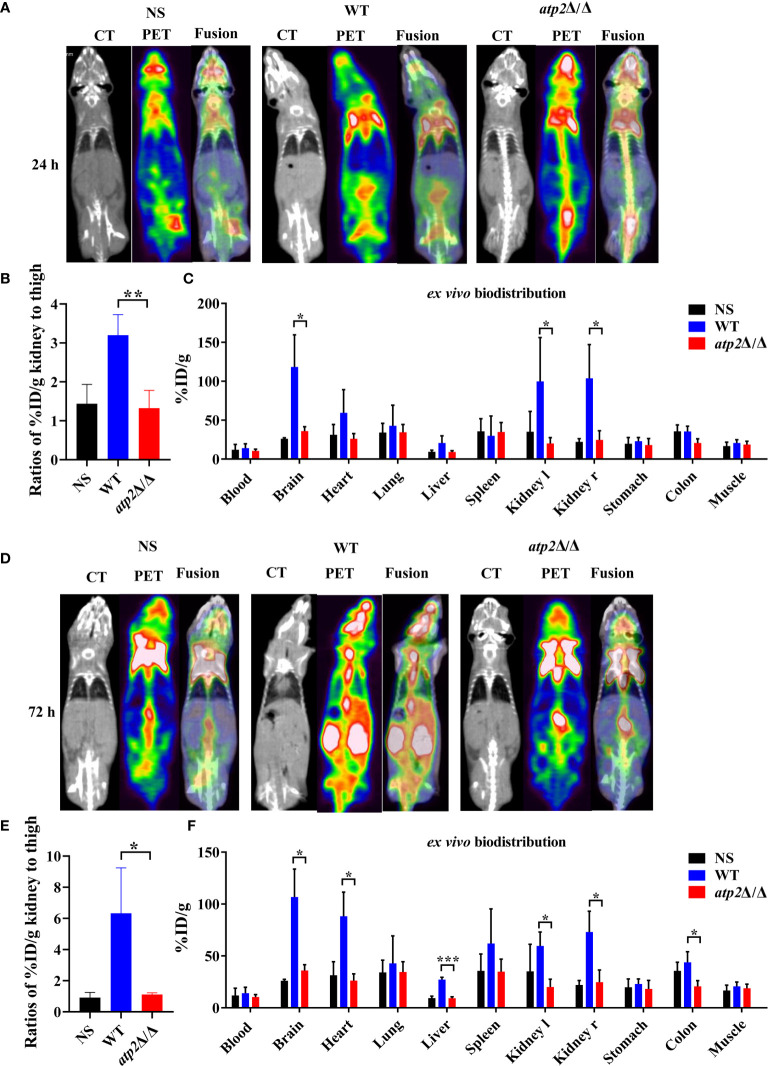
*ATP2* affects the activation of phagocytes by *C. albicans in situ.* **(A, B)** Representative CT, PET, and fusion images **(A)** and quantitative statistics of kidney signals from PET images **(B)** of mice (*n* = 3) 24 h after systemic infection with NS, WT or *atp2*Δ/Δ (2 × 10^5^ CFU per mouse). **(C)** *Ex vivo* biodistribution in different tissues detected by γ-counter immediately after micro-PET-CT examination of **(A)**. **(D, E)** Representative CT, PET, and fusion images **(D)** and quantitative statistics of kidney signals from PET images **(E)** of mice 72 h after systemic infection. **(F)** *Ex vivo* biodistribution in different tissues detected by γ-counter immediately after microPET/CT examination of **(E)**. In **(A, D)**, one representative experiment of three independent experiments is shown. In **(B, C, E, F)**, data are shown as mean ± s.d. *P < 0.05, **P < 0.01, ***P < 0.001; by Student’s *t*-test.

The clearance of pathogens typically requires macrophages to recruit large numbers of phagocytes, including neutrophils and NK cells, to enhance the killing efficiency (Ngo et al., 2014; Netea et al., 2015). Is the efficient clearance of *atp2*Δ/Δ associated with increased recruitment of phagocytes? To answer this question, we injected mice with [^18^F]FDG, which accumulates in activated macrophages and neutrophils, and detected the [18F]FDG signal distribution by microPET/CT and γ counter in real time, dynamically and overall (Davis et al., 2009; Rolle et al., 2016). Surprisingly, the *atp2*Δ/Δ-infected mice never showed an increase in the [18F]FDG signal intensity throughout the body, and there was no significant difference between the *atp2*Δ/Δ and NS groups (**Figures 3A–D**) (*P* > 0.05). However, the [18F]FDG intensity in the WT-infected mice was significantly increased, especially in the target organ, i.e., kidney, where the signal intensity reached 7-fold of that observed in the *atp2*Δ/Δ and NS groups at 72 h (**Figures 3E**) (*P* < 0.05). The brain, heart, liver, and colon signals in the WT group were also stronger than those in the *atp2*Δ/Δ and NS groups (**Figures 3C, F**) (*P* < 0.05).

**Figure 4:**
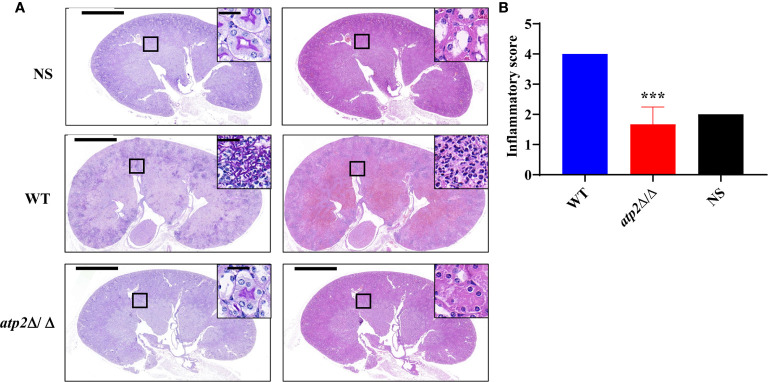
*C. albicans* cells lacking *ATP2* do not cause abnormal phagocytes infiltration in the kidney. **(A)** Representative images of Periodic acid-Schiff-stained (PAS) and Hematoxylin-Eosin-stained (HE) kidney sections of mice (*n* = 3) 72 h after systemic infection with NS, WT or *atp2*Δ/Δ (2 × 10^5^ CFU per mouse). **(B)** Inflammatory score based on renal immune cell infiltration and tissue destruction. In **(A)**, one representative experiment of three mice is shown. In **(B)** data are shown as mean ± s.d. ***P < 0.001; by Student’s *t*-test. In **(A)**, insets show higher-magnification images of boxed areas; scale bars, 1,000 µm, 50 µm (insets).

In alignment with the PET results, we did not observe significant inflammatory foci in the kidney sections from the *atp2*Δ/Δ-infected mice, whereas multiple foci of inflammatory cells containing mostly polymorphonuclear leukocytes (PMNs) were observed in the WT-infected mice (**Figure 4**). The inflammatory scores obtained from renal immune cell infiltration and tissue destruction were significantly lower than those in the WT group (**Figure 4**) (*P* < 0.05). These results suggest that the clearance of the *atp2*Δ/Δ mutant cells did not induce abnormal inflammatory cells recruitment *in situ*.

### *ATP2* Mutant Cells Can Remain Viable in Macrophage-Mimicking Environment

**Figure 5:**
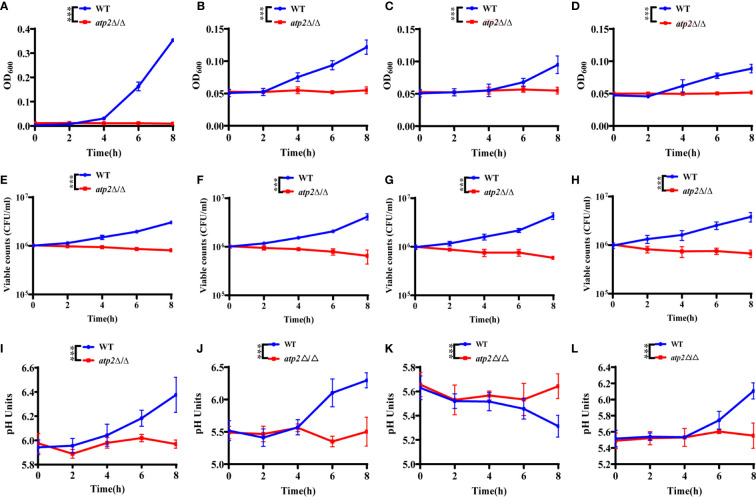
*C. albicans* cells lacking *ATP2* are unable to utilize alternative carbon source. The growth **(A–D)**, viability **(E–H)**, and pH **(I–L)** of WT and *atp2*Δ/Δ cells in YNB medium plus 2% CAA, GLcNAc, oleic acid, and lactate over 8 h. In **(A–L)**, the assays were performed in triplicate.***P < 0.001; by two-way ANOVA.

Once engulfed, *C. albicans* cells rapidly adapt the glucose-deficient environment, shifting to gluconeogenic growth and escaping from macrophages within 6-8 h (Lorenz et al., 2004; Wartenberg et al., 2014). To observe the effect of *ATP2* on the adaptation of *C. albicans* to the environment, we first examined the growth and viability of WT and *atp2*Δ/Δ in the media that simulated glucose-deficient environment within macrophages. The results showed that the OD_600_ of WT group in macrophage-mimicking media (YNB medium with 2% CAA, GlcNAc, lactate, or oleic acid) increased, while the OD_600_ of *atp2*Δ/Δ group remained at the initial level (**Figures 5A–D**). More importantly, consistent with the growth results detected by OD_600_, the viable cells of WT were increased in these macrophage-mimicking media over time, reaching 3-5 times the initial value at 8 h. However, the number of viable *atp2*Δ/Δ in these media decreased by 20-40% at 8 h (**Figures 5E–H**). These results suggest that the *atp2*Δ/Δ cells stop proliferating, but can partially remain viable for a short period of time in the glucose-deficient environment.

*C. albicans* can also adapt to or even alkalize the acidic environment within macrophages, creating more favorable conditions for its survival and escape (Vylkova and Lorenz, 2014). We examined the effect of WT and *atp2*Δ/Δ on the pH value of the environment in macrophage-mimicking media. The results showed that the pH values of the CAA (**Figure 5I**), GlcNAc (**Figure 5J**) and lactate (**Figure 5L**) medium increased simultaneously with the OD_600_ when the WT cells were cultured. The pH of the oleic acid medium gradually decreased when the oleic acid decomposed by WT cells (**Figure 5K**). In contrast, the pH values of the media with *atp2*Δ/Δ cells were not statistically different at each time point, the *atp2*Δ/Δ cells were unable to change the environment pH in all macrophage-mimicking media (**Figures 5I–K**). The above results suggest that deletion of *ATP2* impaired the adaptation of *C. albicans* to the glucose-deficient environment within macrophages.

### *ATP2* Mutant Cells Are Unable to Undergo Morphogenesis in Macrophages

**Figure 6:**
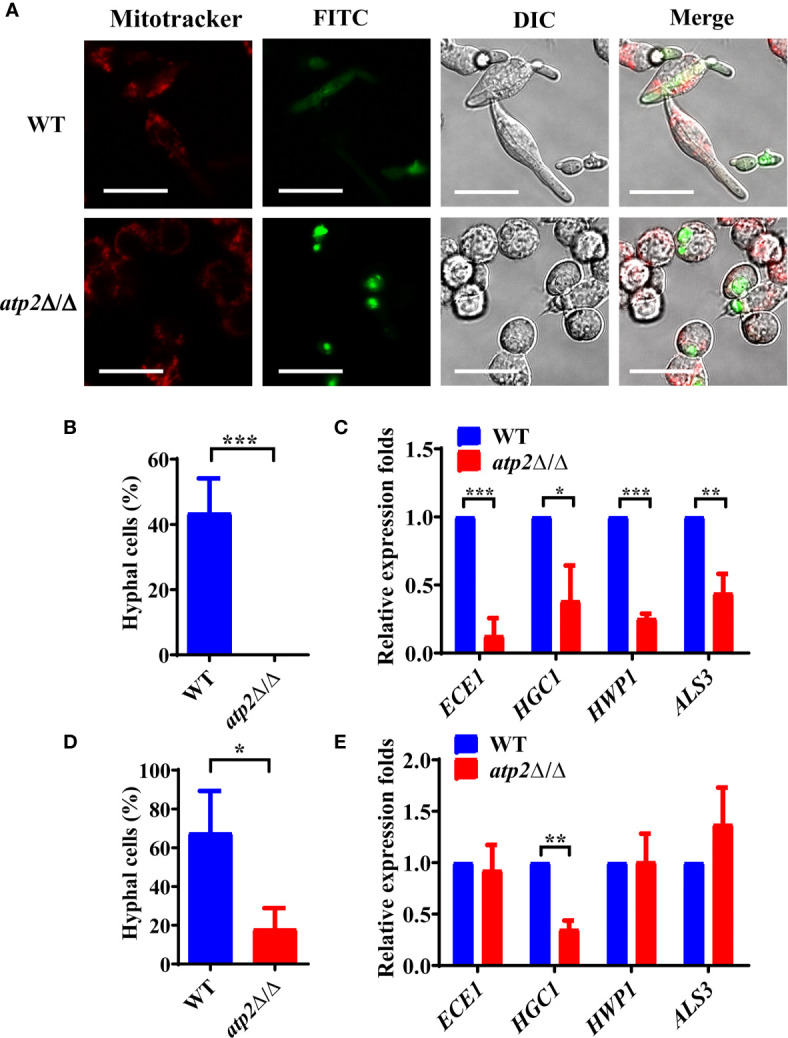
Hyphae formation of *C. albicans* inside and outside of the macrophages. **(A)** Representative images of FITC-stained WT and *atp2*Δ/Δ cells (green) co-cultured with MitoTracker Deep Red-preloaded RAW264.7 (red) at a 1:1 ratio for 3 h. Images were obtained by confocal laser scanning microscopy at channels E_x644_/E_m655_, E_x488_/E_m525_ and DIC, ×630. **(B, D)** The percentage of hyphal cells inside **(B)** and outside **(D)** macrophages was calculated from 50 cells each group. **(C, E)** The relative expression level of hyphal-related genes in WT and *atp2*Δ/Δ inside **(C)** and outside **(E)** macrophages after cocultured for 5 h. In **(A)**, one representative experiment of three independent experiments is shown; scale bars, 20 µm. In **(B–E)** data are shown as mean ± s.d. *P < 0.05, **P < 0.01, ***P < 0.001; by Student’s *t*-test.

Morphogenesis is an important contributing factor for the escape from macrophages (Peroumal et al., 2019; Rogiers et al., 2019). To investigate the hyphae formation of these viable *atp2*Δ/Δ cells, we observed the status of FITC-stained *C. albicans* (green) cocultured with MitoTraker-preloaded macrophages (red) when cocultured for 3 h. The results showed that WT cells completed morphogenesis with a germination rate of 45.4%, whereas the *atp2*Δ/Δ mutant cells were trapped inside macrophages in the yeast phase (**Figures 6A, B**). Then, we extracted RNA from phagocytic *C. albicans* to conduct RT-qPCR and found that the expression levels of the genes involved in hyphal formation (*ECE1, HGC1, HWP1* and *ALS3*) were significantly lower than those in the WT group (*P* < 0.05) (**Figure 6C**).

Interestingly, we found that the unengulfed *atp2*Δ/Δ mutant could undergo morphogenesis in the medium with a germination rate of 18.1% (**Figure 6D**). Under this condition, only *HGC1* was expressed at a significantly lower level than that in the WT, while the expression levels of *ECE1, HWP1* and *ALS3* did not significantly differ (**Figure 6E**). The above results suggest that deletion of *ATP2* prevents *C. albicans* from morphogenesis inside macrophages, but not outside of them.

### Morphogenesis of *ATP2* Mutant Cells Are Affected by the Presence or Absence of Glucose

**Figure 7:**
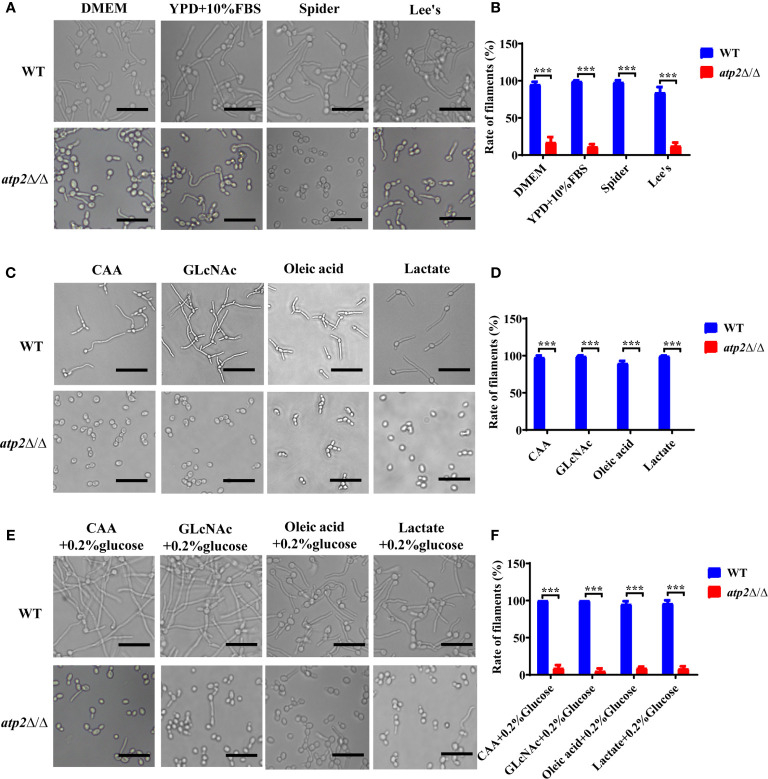
Hyphae formation of *C albicans* in various environments. **(A, B)** Representative images and hyphal cell percentages of WT and *atp2*Δ/Δ cultured in DMEM, YPD+10% FBS, Spider, and Lee’s medium at 37°C for 3 h. **(C, D)** Representative images and hyphal cell percentages of WT and *atp2*Δ/Δ cultured in CAA, GLcNAc, oleic acid, and lactate medium at 37°C for 3 h. **(E, F)** Representative images and hyphal cell percentages of WT and *atp2*Δ/Δ cultured in CAA, GLcNAc, oleic acid, and lactate medium supplemented with 0.2% glucose at 37°C for 3 h. In **(B, D, F)** the percentage of hyphal cells in each medium was calculated from at least 100 cells. The assays were performed in triplicate. ***P < 0.001; by Student’s *t*-test.

Why *atp2*Δ/Δ cells were unable to form hyphae in macrophages? We performed hyphae formation assay in classic hyphae induction media (YPD+10% FBS, Spider, Lee’s), DMEM, and macrophage-mimicking media. The results showed that more than 90% of the WT cells formed elongated hyphae in all the media mentioned above, while the *atp2*Δ/Δ cells only formed short hyphae in media containing glucose (DMEM, YPD+10% FBS, and Lee’s) with germination rates around 10% (**Figures 7A–D**), and could not reach a level close to that of WT even with extended incubation time (**Figure S2**). In Spider medium (no glucose inside) and macrophage-mimicking media, the *atp2*Δ/Δ cells didn’t form hyphae (**Figures 7A–D**). Then we supplemented macrophage-mimicking media with 0.2% glucose, which was close to glucose level in blood, and found that hyphae formation of *atp2*Δ/Δ was partially restored (**Figures 7E, F**). The above results suggest that the hyphae formation of *atp2*Δ/Δ is reduced, but whether it forms hyphae also related to the presence or absence of glucose.

### *ATP2* Mutant Cells Are More Susceptible to Oxidative Stress

**Figure 8:**
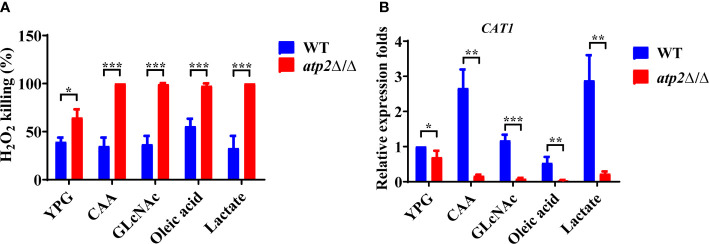
*C albicans* cells lacking *ATP2* are more vulnerable to oxidative stress in macrophage-mimicking media. **(A)** The percentage of *C albicans* cells killed by 6 mM H_2_O_2_ in YNB medium plus 2% glucose, CAA, GLcNAc, oleic acid, and lactate at 6 h. The killing rate was determined by comparison with *C albicans* recovered without H_2_O_2_. **(B)** Relative expression levels of *CAT1* in different media, and the expression level of WT in YNB + 2% glucose was set as 1. In **(A, B)**, data are shown as mean ± s.d. *P < 0.05, **P < 0.01, ***P < 0.001; by Student’s *t*-test.

Oxidative stress within the phagosome creates a toxic environment that induces oxidative stress and programmed cell death in *C. albicans* (Dantas Ada et al., 2015). We further investigated the resistance of *atp2*Δ/Δ to oxidative stress (6 mM H_2_O_2_) in different environments. The results showed that 39.3% and 64.7% of the WT and *atp2*Δ/Δ cells were killed in the glucose medium at 6 h, respectively (*P* < 0.05) (**Figure 8A**). However, in the macrophage-mimicking media mentioned above, almost all *atp2*Δ/Δ cells were killed under the same H_2_O_2_ concentration, while the WT cells exhibited only slightly increased sensitivity to H_2_O_2_ in oleic acid (**Figure 8A**).

We further tested the transcription levels of the *CAT1* gene (which protects *C. albicans* from oxidative stress) in the *atp2*Δ/Δ and WT cells under different conditions (Dantas Ada et al., 2015). Consistent with the phenotype, the expression level of *CAT1* in the *atp2*Δ/Δ cells was decreased by 31.6% in the glucose medium compared with that in the WT (**Figure 8B**). However, the *CAT1* levels in the *atp2*Δ/Δ cells were greatly downregulated in the alternative carbon source but were upregulated (CAA and lactate), unchanged (GlcNAc), or slightly downregulated (oleic acid) in the WT cells (*P* < 0.05) (**Figure 8B**). It can be seen that *atp2*Δ/Δ cells are more susceptible to oxidative stress, especially in the glucose-deficient environments.

### Deletion of *ATP2* in *C. albicans* Affects Proteins Involved in Its Escape From Macrophages

To further investigate the effect of *ATP2* on multiple abilities associated with *C. albicans* escape from macrophages, we performed a proteomics study and found that deletion of *ATP2* resulted in 112 proteins up-regulated and 268 proteins down-regulated (*P* < 0.05 and fold change > 1.5). Here, we focused on proteins involved in hyphae formation, stress responses, alternative carbon source utilization, etc.

**Figure 9:**
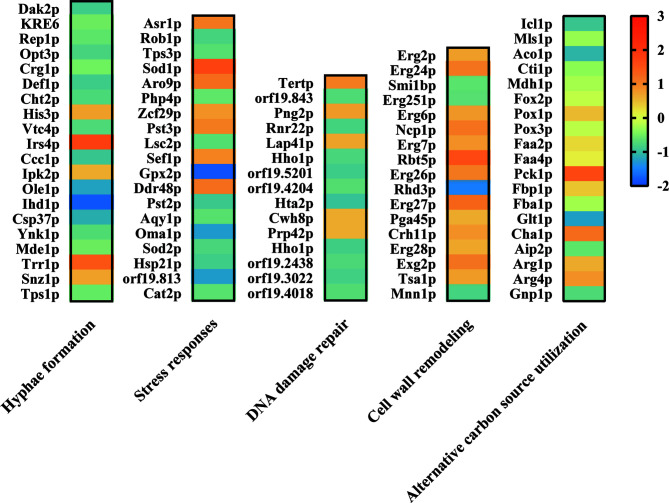
Effect of *ATP2* on proteins associated with *C. albicans* escape from macrophage. Summary of differentially expressed proteins related to hyphae formation, stress responses, DNA damage repair, cell wall remodeling, and alternative carbon source utilization in proteomic results. Compared to WT, the differentially expressed proteins in *atp2*Δ/Δ mutant were identified at *p* < 0.05 and fold change > 1.5. The log_2_(FC) values were presented as a heat map.

When referred to the WT strain, several proteins required for hyphae formation were down-regulated, for example Ihd1p, Opt3p, and Ole1p were 0.21-, 0.54-, and 0.35-fold down-regulated in the *atp2*Δ/Δ mutant (**Figure 9**). In addition, putative glutathione peroxidase (Gpx2p) involved in Cap1p-dependent oxidative stress response was 0.28-fold down-regulated in *atp2*Δ/Δ mutant (**Figure 9**). However, Sod1p, which play a protective role against oxidative stress, was upregulated 3.3-fold in *atp2*Δ/Δ mutant (**Figure 9**). Proteins related to cell wall remodeling, GPI-anchored cell wall proteins (Exg2p, Rbt5p, and Crh11p) were up-regulated in *atp2*Δ/Δ mutant. Proteins associated with DNA damage repair were generally down-regulated.

As for proteins involved in alternative carbon source utilization, all enzymes (Icl1p, Mls1p, Aco1p, Cti1p, and Mdh1p) involved in the GC pathway were significantly repressed in the *atp2*Δ/Δ cells, two of the eight (Pck1p and Fbp1p) gluconeogenesis enzymes were upregulated, and there was no significant change in the β-oxidation pathway (**Figure 9**). The proteomic results were generally consistent with the phenotype, proteins related to multiple functions related to *C. albicans* escape from macrophages were repressed to some extent.

## Discussion

The ability of *C. albicans* to persist in the human host and cause disease requires the capacity to evade and circumvent host defense mechanisms, especially macrophage-mediated clearance (O’Meara et al., 2018; Austermeier et al., 2020). Therefore, the *C. albicans*-macrophage interaction process offers promising targets for much-needed novel therapeutics to treat fungal infections and has not been exploited as a therapeutic target to date.

We found that the *ATP2* deletion increased the macrophage clearance of *C. albicans* from 30.8% to 98.8%, almost completely preventing the escape of *C. albicans*. However, not only *ATP2*, there are other genes affecting the escape of *C. albicans* from macrophages. When calculated the variations in clearance rate using their parental strains as standard, the deletion of *PHO4* (78%), *TRK1* (70%), *ALI1* (65.5%), or *CYR1* (64.1%) caused changes in macrophage clearance rates of similar magnitude compared to *ATP2* (67.4%) (**Table S2**) (Rocha et al., 2001; Ikeh et al., 2016; Llopis-Torregrosa et al., 2019; Williams and Lorenz, 2020). When directly comparing the percentage of killed mutant cells, the deletion of *ALI1, CYR1, RTT109, COX4*, or *RAS1* increased the clearance of *C. albicans* to more than 90% (98.2% of the *atp2*Δ/Δ) (**Table S2**) (Marcil et al., 2002; Lopes da Rosa et al., 2010; Williams and Lorenz, 2020). Although different macrophage types, co-culture times, MOIs can lead to differences in the data from one study to another, it can provide us with some reference information when all mutants are not available for simultaneous experiments. Through this analysis, we found that *ATP2*, as well as two other genes related to mitochondrial function (*ALI1* and *CYR1*) were at the forefront of the genes currently known to affect clearance.

The *ATP2* mutant also displayed a reduced ability to escape macrophage clearance *in vivo*. We constructed macrophage-depleted mice by clodronate liposome, a simple, and stable method that has been widely used to study macrophage-pathogen interactions (Wirnsberger et al., 2016; Moreno, 2018). The persist of *atp2*Δ/Δ in macrophage-depleted mice suggested that it could survive, if not grow, in organs. Our results of growth in tissue homogenates supported this conclusion (**Table S3**). Thus, under the premise that *atp2*Δ/Δ could survive and even grow *in vivo*, the results of *atp2*Δ/Δ were undetectable in various organs of macrophage-normal mice suggesting that the mutant strain had a reduced ability to escape macrophage clearance *in vivo*.

Most *C. albicans* wild type cells can escape from the macrophage in 6-8 h (Wartenberg et al., 2014). In the early phase (1 h) of *C. albicans-*macrophages interaction, *C. albicans* switched to a slow gluconeogenic growth mode in the glucose-deficient environment, and in the late phase (6-8 h), along with hyphae formation and escape, *C. albicans* resumed rapid glycolytic growth (Lorenz et al., 2004; Tucey et al., 2018; Laurian et al., 2020). Why did *ATP2* deletion lead to a substantial reduction of *C. albicans* escaping macrophage clearance? According to our results, *atp2*Δ/Δ cells had good glycolytic growth but were unable to undergo gluconeogenic growth. However, even if the mutant failed to proliferate, 60-80% of *atp2*Δ/Δ cells could remained viable for 8 h in glucose-deficient environments. Our previous work reported that viability of *atp2*Δ/Δ decreased substantially in glucose-deficient media after 24 h, but it could survive and proliferate in glucose-sufficient media (Li et al., 2018). Therefore, the ability to escape macrophages and resume glycolytic growth within 8 h is critical for *atp2*Δ/Δ to survive *C. albicans-*macrophages interaction.

Hyphal morphogenesis is a key factor promoting *C. albicans* escape from macrophages in a physical or inflammation dependent manner (Peroumal et al., 2019; Rogiers et al., 2019). We observed that *atp2*Δ/Δ cells were all trapped within macrophages in the yeast form. However, when we studied the morphogenesis ability of *atp2*Δ/Δ we found that the hyphae formation of the mutant was reduced but not absent in media containing glucose, and the expression level of related genes (*ECE1, HWP1* and *ALS3*) was not even statistically different from WT. Therefore, the inability of *atp2*Δ/Δ cells to form hyphae within macrophages was likely due to their fitness defect rather than specific hyphae formation defect.

Unlike specific hyphae formation or carbon source utilization genes, *ATP2* has a broader effect on the function involved in *C. albicans*-macrophages interaction. Specific hyphae formation defective mutant (*cph1*Δ/*efg1*Δ) was unable to escape from macrophages after 24 h, but no other functional defects allowed these cells to survive and still replicate in the yeast form (Wartenberg et al., 2014). Moreover, the sustained interaction of *cph1*Δ/*efg1*Δ with macrophages may lead to new variants capable of escape from macrophage (Wartenberg et al., 2014). Due to the broad effect of *ATP2* on the ability of *C. albicans* associated with escape from macrophage, *C. albicans* cannot bypass or tolerate its inhibitory effects, rendering it a better target than specific functionally related genes.

Since F_1_F_o_-ATP synthase is evolutionarily conserved in bacteria, fungi, and mammals, and using the β subunit (encoded by *ATP2*) or other subunits of F_1_F_o_-ATP synthase as drug target may carry a risk of toxicity (Jonckheere et al., 2012). Bedaquiline is an FDA-approved anti-tuberculosis drug that targets the c-ring of F_1_F_o_-ATP synthase, which is 20,000 times more sensitive to *Mycobacterium tuberculosi*s than to mammal cells (Andries et al., 2005; Fiorillo et al., 2016). Although the β subunit has not yet been used as an anti-infection drug target, some small molecules and monoclonal antibodies targeting the β subunit have entered phase I or II clinical trials in antitumor studies, such as angiostatin, Hai178, and Aurovertin B, all of which have shown selective inhibition of tumor cells with low toxicity to normal cells (Moser et al., 2001; Huang et al., 2008; Chen et al., 2016).

In summary, deletion of *ATP2* prevents *C. albicans* from escaping macrophage clearance *in vitro* and *in vivo*. *ATP2* has the functional basis as a drug target of *C. albicans-* macrophages interaction and deserves further investigation in the future.

## Data Availability Statement

The proteomics data presented in the study were deposited in the ProteomeXchange Consortium, accession number was PXD024039.

## Ethics Statement

The animal study was reviewed and approved by Laboratory Animal Ethics Committee of Jinan University.

## Author Contributions

YiZ, CT, and ZZ. contributed equally to the article. YiZ and SL developed the concept and designed the research plan. YiZ, CT, ZZ, YaZ, and LW performed experiments. YiZ, ZZ, and CT performed statistical analysis. YiZ, ZZ, and CT wrote the paper. All authors contributed to the article and approved the submitted version.

## Funding

This work was supported by the National Natural Science Foundation of China (81971913/81471995/81903675) and China Postdoctoral Science Foundation (2019M653291).

## Conflict of Interest

The authors declare that the research was conducted in the absence of any commercial or financial relationships that could be construed as a potential conflict of interest.
