# Supplementary material for: Deletion of the ATP2 Gene in Candida albicans Blocks Its Escape From Macrophage Clearance
Source: Front Cell Infect Microbiol. 2021 Apr 16;11:643121. doi: 10.3389/fcimb.2021.643121 (PMC8085345; doi:10.3389/fcimb.2021.643121)
Supplement: Supplementary file 1 [file DataSheet_1.docx]

**Supplementary Material**

**Supplementary tables**

| Primer | Sequence (5'-3') | Reference |
| --- | --- | --- |
| HWP1-F | TGTCTACACTACATTCTGTC | (Ding et al., 2013) |
| HWP1-R | AGGAATAGATGGTTGTGAAC | (Ding et al., 2013) |
| ECE1-F | CCAAGCACCTACTGTTCC | (Ding et al., 2013) |
| ECE1-R | GATACCAGCAACAACAGAAT | (Ding et al., 2013) |
| ALS3-F | CTCATTACACCAACCATACA | (Ding et al., 2013) |
| ALS3-R | GGATTCTGTGGTTGTAGTAT | (Ding et al., 2013) |
| HGC1-F | GTATCGCTGGTTCTCGTGCT | (Li et al., 2017a) |
| HGC1-R | AGGTGTACCACTACCACCATT | (Li et al., 2017a) |
| CAT1-F | GAGACCCATCTAAATTCCCAC | (Tscherner et al., 2015) |
| CAT1-F | CTTCATTGCTAGTCAAGTAATCCC | (Tscherner et al., 2015) |
| 18S-F | CGCAAGGCTGAAACTTAAAGG | (Tscherner et al., 2015) |
| 18S-R | AGCAGACAAATCACTCCACC | (Tscherner et al., 2015) |

**Table S1** Primers used in this study

**Table S2** *C. albicans* genes that affect macrophage clearance after deletion

| Gene | Clearance  of mutant  (%) | Clearance of WT  (%) | Coculture time | Macrophage: *C.albicans* | Reference |  |
| --- | --- | --- | --- | --- | --- | --- |
| *PHO4* | 80 | 2 | 6 h | 1:3 | (Ikeh et al., 2016) |  |
| *TRK1* | 40 | -30 | 24 h | 1:1 | (Llopis-Torregrosa et al., 2019) |  |
| *ALI1* | 98 | 32.5 | 16 h | 5:1 | (Williams and Lorenz, 2020) |  |
| *CYR1* | 95.5 | 31.4 | 48 h | 2.5:1 | (Rocha et al., 2001) |  |
| *COX4* | 92 | 32.5 | 16 h | 5:1 | (Williams and Lorenz, 2020) |  |
| *SIN3* | 80 | 32.5 | 16 h | 5:1 | (Williams and Lorenz, 2020) |  |
| *OAR1* | 78 | 32.5 | 16 h | 5:1 | (Williams and Lorenz, 2020) |  |
| *ATP1* | 58 | 14 | 12 h | 1:2.5 | (Li et al., 2017b) |  |
| *NGT1* | 80 | 37.5 | 48 h | 2.5:1 | (Vesely et al., 2017) |  |
| *GPX31* | 40 | 0 | 48 h | 1:1 | (Miramón et al., 2014) |  |
| *STP2* | 55 | 16 | 48 h | 2.5:1 | (Danhof and Lorenz, 2015) |  |
| *ERG11* | 38 | - | 48 h | 3:1 | (Wu et al., 2018) |  |
| *ATO1* | 52 | 16 | 48 h | 2.5:1 | (Danhof and Lorenz, 2015) |  |
| *SWI4* | 65 | 32.5 | 16 h | 5:1 | (Williams and Lorenz, 2020) |  |
| *ATO5* | 48 | 16 | 48 h | 2.5:1 | (Danhof and Lorenz, 2015) |  |
| *RTT109* | 93 | 62.5 | 16 h | 15:1 | (Lopes da Rosa et al., 2010) |  |
| *GPI2* | 54 | 25.8 | 18 h | 1:5 | (Jain et al., 2018) |  |
| *CPH1* | 60 | 32.5 | 16 h | 5:1 | (Williams and Lorenz, 2020) |  |
| *PEP8* | 60 | 32.5 | 16 h | 5:1 | (Williams and Lorenz, 2020) |  |
| *TPS1* | 45 | 17.5 | 2 h | 1:10 | (Martínez-Esparza et al., 2011) |  |
| *PEX13* | 58 | 32.5 | 16 h | 5:1 | (Williams and Lorenz, 2020) |  |
| *GCN5* | 85 | 60 | 2 h | 1:10 | (Shivarathri et al., 2019) |  |
| *ICL1* | 70 | 45 | 48 h | 1:5 | (Childers et al., 2016) |  |
| *KIS1* | 55 | 32.5 | 16 h | 5:1 | (Williams and Lorenz, 2020) |  |
| *RAS1* | 90.1 | 67.8 | 48 h | 3:5 | (Marcil et al., 2002) |  |
| *CWT1* | 35 | 15 | 24 h | 1:2 | (Danhof et al., 2016) |  |
| *GPR1* | 30 | 10 | 3 h | 1:1 | (Maidan et al., 2008) |  |
| *ORF19.6688* | 40 | 22 | 48 h | - | (Wilson et al., 2014) |  |
| *CST20* | 78 | 67.8 | 48 h | 3:5 | (Marcil et al., 2002) |  |
| *PRA1* | 39.5 | 37.7 | 24 h | 1:1 | (Marcil et al., 2008) |  |
|  | 62.9 | 61.5 | 24 h | 1:1 | (Marcil et al., 2008) |  |

**Table S3** Growth of *C. albicans* in tissue homogenates (10^5^cells/ml)

|  | Liver | Kidney | Spleen | Heart | Brain | Serum |
| --- | --- | --- | --- | --- | --- | --- |
| WT  *atp2*Δ/Δ | 381±45  124.4±14.4 | 84.6±9.6  12.1±1.26 | 87±3  14.9±0.9 | 78±18  0.6±0.6 | 53.1±3.3  0.45±0.21 | 51.3±1.5  13.1±1.2 |

**Supplementary Figures**

**Figure S1** The growth of WT and *atp2*Δ/Δ cells in DMEM without macrophages over 24 h. The assays were performed in triplicate.*, P < 0.05; **, P < 0.01; ***, P < 0.001; by two-way ANOVA.


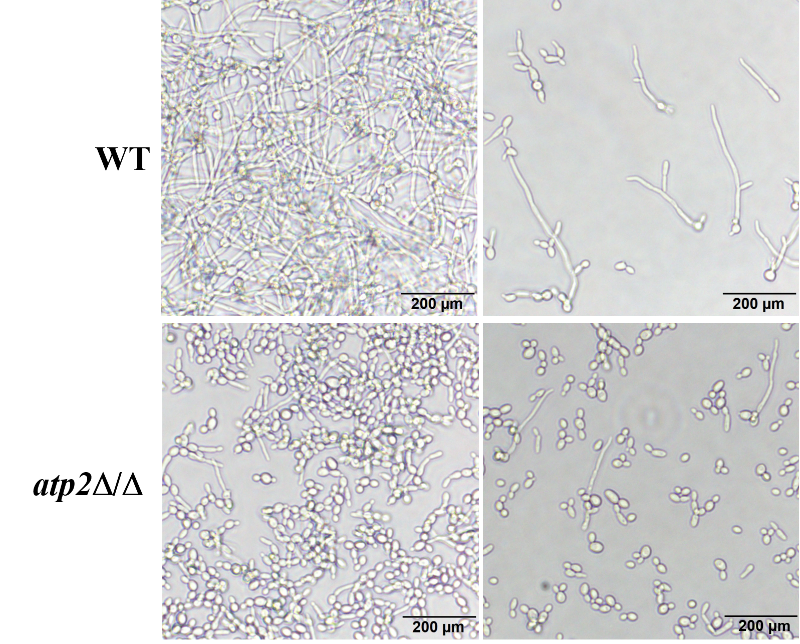


**Figure S2** The hyphae formation of WT and *atp2*Δ/Δ cells in YPD+10%FBS medium incubated at 37 ℃ for 7 h.

**Figure S3** Survival of mice after systemic infection of WT and *atp2*Δ/Δ. Mice were injected with PBS liposomes (macrophage+) or clodronate liposomes (macrophage-) 24 h before and 24 h after intravenous infection with *C. albicans* (2×10^5^ CFU per mouse). Each group includes 8 mice. *, P < 0.05; **, P < 0.01; ***, P < 0.001; by Log-rank (Mantel-Cox) test.

**Supplemental References**

Childers, D.S., Raziunaite, I., Mol Avelar, G., Mackie, J., Budge, S., Stead, D., et al. (2016). The Rewiring of Ubiquitination Targets in a Pathogenic Yeast Promotes Metabolic Flexibility, Host Colonization and Virulence. *PLoS Pathog* 12(4)**,** e1005566. doi: 10.1371/journal.ppat.1005566.

Danhof, H.A., and Lorenz, M.C. (2015). The Candida albicans ATO Gene Family Promotes Neutralization of the Macrophage Phagolysosome. *Infect Immun* 83(11)**,** 4416-4426. doi: 10.1128/iai.00984-15.

Danhof, H.A., Vylkova, S., Vesely, E.M., Ford, A.E., Gonzalez-Garay, M., and Lorenz, M.C. (2016). Robust Extracellular pH Modulation by Candida albicans during Growth in Carboxylic Acids. *mBio* 7(6). doi: 10.1128/mBio.01646-16.

Ding, X., Yu, Q., Xu, N., Wang, Y., Cheng, X., Qian, K., et al. (2013). Ecm7, a regulator of HACS, functions in calcium homeostasis maintenance, oxidative stress response and hyphal development in Candida albicans. *Fungal Genet Biol* 57**,** 23-32. doi: 10.1016/j.fgb.2013.05.010.

Ikeh, M.A., Kastora, S.L., Day, A.M., Herrero-de-Dios, C.M., Tarrant, E., Waldron, K.J., et al. (2016). Pho4 mediates phosphate acquisition in Candida albicans and is vital for stress resistance and metal homeostasis. *Mol Biol Cell* 27(17)**,** 2784-2801. doi: 10.1091/mbc.E16-05-0266.

Jain, P., Sethi, S.C., Pratyusha, V.A., Garai, P., Naqvi, N., Singh, S., et al. (2018). Ras signaling activates glycosylphosphatidylinositol (GPI) anchor biosynthesis via the GPI-N-acetylglucosaminyltransferase (GPI-GnT) in Candida albicans. *J Biol Chem* 293(31)**,** 12222-12238. doi: 10.1074/jbc.RA117.001225.

Li, S.-X., Song, Y.-J., Zhang, Y.-S., Wu, H.-T., Guo, H., Zhu, K.-J., et al. (2017a). Mitochondrial Complex V α Subunit Is Critical for Candida albicans Pathogenicity through Modulating Multiple Virulence Properties. *Frontiers in Microbiology* 8(285). doi: 10.3389/fmicb.2017.00285.

Li, S.X., Song, Y.J., Zhang, Y.S., Wu, H.T., Guo, H., Zhu, K.J., et al. (2017b). Mitochondrial Complex V α Subunit Is Critical for Candida albicans Pathogenicity through Modulating Multiple Virulence Properties. *Front Microbiol* 8**,** 285. doi: 10.3389/fmicb.2017.00285.

Llopis-Torregrosa, V., Vaz, C., Monteoliva, L., Ryman, K., Engstrom, Y., Gacser, A., et al. (2019). Trk1-mediated potassium uptake contributes to cell-surface properties and virulence of Candida glabrata. *Sci Rep* 9(1)**,** 7529. doi: 10.1038/s41598-019-43912-1.

Lopes da Rosa, J., Boyartchuk, V.L., Zhu, L.J., and Kaufman, P.D. (2010). Histone acetyltransferase Rtt109 is required for Candida albicans pathogenesis. *Proc Natl Acad Sci U S A* 107(4)**,** 1594-1599. doi: 10.1073/pnas.0912427107.

Maidan, M.M., De Rop, L., Relloso, M., Diez-Orejas, R., Thevelein, J.M., and Van Dijck, P. (2008). Combined inactivation of the Candida albicans GPR1 and TPS2 genes results in avirulence in a mouse model for systemic infection. *Infect Immun* 76(4)**,** 1686-1694. doi: 10.1128/iai.01497-07.

Marcil, A., Gadoury, C., Ash, J., Zhang, J., Nantel, A., and Whiteway, M. (2008). Analysis of PRA1 and its relationship to Candida albicans- macrophage interactions. *Infect Immun* 76(9)**,** 4345-4358. doi: 10.1128/iai.00588-07.

Marcil, A., Harcus, D., Thomas, D.Y., and Whiteway, M. (2002). Candida albicans killing by RAW 264.7 mouse macrophage cells: effects of Candida genotype, infection ratios, and gamma interferon treatment. *Infect Immun* 70(11)**,** 6319-6329. doi: 10.1128/iai.70.11.6319-6329.2002.

Martínez-Esparza, M., Tapia-Abellán, A., Vitse-Standaert, A., García-Peñarrubia, P., Argüelles, J.C., Poulain, D., et al. (2011). Glycoconjugate expression on the cell wall of tps1/tps1 trehalose-deficient Candida albicans strain and implications for its interaction with macrophages. *Glycobiology* 21(6)**,** 796-805. doi: 10.1093/glycob/cwr007.

Miramón, P., Dunker, C., Kasper, L., Jacobsen, I.D., Barz, D., Kurzai, O., et al. (2014). A family of glutathione peroxidases contributes to oxidative stress resistance in Candida albicans. *Med Mycol* 52(3)**,** 223-239. doi: 10.1093/mmy/myt021.

Rocha, C.R., Schröppel, K., Harcus, D., Marcil, A., Dignard, D., Taylor, B.N., et al. (2001). Signaling through adenylyl cyclase is essential for hyphal growth and virulence in the pathogenic fungus Candida albicans. *Mol Biol Cell* 12(11)**,** 3631-3643. doi: 10.1091/mbc.12.11.3631.

Shivarathri, R., Tscherner, M., Zwolanek, F., Singh, N.K., Chauhan, N., and Kuchler, K. (2019). The Fungal Histone Acetyl Transferase Gcn5 Controls Virulence of the Human Pathogen Candida albicans through Multiple Pathways. *Sci Rep* 9(1)**,** 9445. doi: 10.1038/s41598-019-45817-5.

Tscherner, M., Zwolanek, F., Jenull, S., Sedlazeck, F.J., Petryshyn, A., Frohner, I.E., et al. (2015). The Candida albicans Histone Acetyltransferase Hat1 Regulates Stress Resistance and Virulence via Distinct Chromatin Assembly Pathways. *PLoS Pathog* 11(10)**,** e1005218. doi: 10.1371/journal.ppat.1005218.

Vesely, E.M., Williams, R.B., Konopka, J.B., and Lorenz, M.C. (2017). N-Acetylglucosamine Metabolism Promotes Survival of Candida albicans in the Phagosome. *mSphere* 2(5). doi: 10.1128/mSphere.00357-17.

Williams, R.B., and Lorenz, M.C. (2020). Multiple Alternative Carbon Pathways Combine To Promote Candida albicans Stress Resistance, Immune Interactions, and Virulence. *mBio* 11(1). doi: 10.1128/mBio.03070-19.

Wilson, D., Mayer, F.L., Miramón, P., Citiulo, F., Slesiona, S., Jacobsen, I.D., et al. (2014). Distinct roles of Candida albicans-specific genes in host-pathogen interactions. *Eukaryot Cell* 13(8)**,** 977-989. doi: 10.1128/ec.00051-14.

Wu, Y., Wu, M., Wang, Y., Chen, Y., Gao, J., and Ying, C. (2018). ERG11 couples oxidative stress adaptation, hyphal elongation and virulence in Candida albicans. *FEMS Yeast Res* 18(7). doi: 10.1093/femsyr/foy057.
